# Establishing the reference broth microdilution MIC method for cefepime-taniborbactam

**DOI:** 10.1128/jcm.00661-25

**Published:** 2025-08-19

**Authors:** Adam Belley, Susan M. Cusick, Dan C. Pevear, Laura Koeth, Jeanna DiFranco-Fisher, Nimmi Kothari, Stephen Hawser, Greg Moeck

**Affiliations:** 1 Venatorx Pharmaceuticals Inc 540451 Malvern, Pennsylvania, USA; 2 Laboratory Specialists Inc 734731 Westlake, Ohio, USA; 3 International Health Management Associates Europe SàrlMonthey, Switzerland; Johns Hopkins University, Baltimore, Maryland, USA

**Keywords:** carbapenemase, *Pseudomonas aeruginosa*, enterobacterales, taniborbactam, cefepime, susceptibility testing, broth microdilution, reference method

## Abstract

The investigational β-lactam/β-lactamase inhibitor combination cefepime-taniborbactam is intended as therapy for serious infections caused by Gram-negative pathogens resistant to third-generation cephalosporins and carbapenems. Establishing a susceptibility testing reference method for cefepime-taniborbactam that conforms to the Clinical Laboratory Standards Institute (CLSI) M07 and International Standards Organization 20776-1:2019 standards is necessary to inform patient care. This study describes the reference broth microdilution MIC method for cefepime-taniborbactam (taniborbactam fixed at 4 µg/mL). In a CLSI M23 Tier 2 study that included nine clinical microbiology laboratories, the CTX-M-15-producer *Escherichia coli* NCTCC 13353 was determined to be appropriate for routine quality control (QC) as ranges for cefepime (≥64 µg/mL) and cefepime-taniborbactam (0.12 to 1 µg/mL) were non-overlapping, thereby simultaneously controlling for cefepime antibacterial activity and taniborbactam β-lactamase inhibition. Of the cefepime-taniborbactam MIC results obtained, 99.6% (269/270) were within the identified QC range; similarly, 100% (79/79) and 98.0% (98/100) of the QC values from a surveillance study and from the two central microbiology laboratories of the CERTAIN-1 Phase 3 clinical study (clintrials.gov identifier NCT03840148) were in range, respectively. Modifications to the standard medium (pH, cation content, or supplementation with human serum, albumin, polysorbate-80, or pulmonary surfactant) or assay parameters (inoculum density, incubation duration, and atmosphere) revealed that only inoculum titers (e.g., ≥5 × 10^6^ CFU/mL) exceeding the CLSI M07 standard (2–8 × 10^5^ CFU/mL) increased MIC values above the QC range. These results demonstrate the robustness and reliability of the cefepime-taniborbactam broth microdilution MIC reference method when performed following the approved standards.

**IMPORTANCE:**

This study focuses on a new antibiotic combination called cefepime-taniborbactam that is being developed to treat serious infections caused by bacteria that are often resistant to current treatments. To make sure this new antibiotic combination can be used safely and effectively once it has been approved for clinical use, we developed a standardized laboratory method to measure its activity against certain bacteria that are widely used during quality control testing. The method was assessed in multiple labs and proved to be reliable, accurate, and consistent. It also held up well under different testing conditions, showing that it is a dependable tool for guiding treatment decisions. This is an important step in meeting the challenge of antibiotic-resistant infections since it will help clinicians evaluate cefepime-taniborbactam as a potential treatment option as they strive to improve the care of patients suffering from serious infections.

## INTRODUCTION

The evolution of β-lactam resistance in Gram-negative pathogens continues to alter the treatment landscape for hospital-acquired infections (1–3). The increased prevalence of extended-spectrum β-lactamases (ESBL) and resistance to carbapenems through the acquisition of *Klebsiella pneumoniae* carbapenemase (KPC), metallo-β-lactamases (MBL; e.g., NDM and VIM), or the oxacillinase OXA-48 has necessitated the need for new therapies to treat third-generation cephalosporin-resistant Enterobacterales, carbapenem-resistant Enterobacterales, and difficult-to-treat *Pseudomonas aeruginosa* (4). Developing new β-lactamase inhibitors that target these resistance mechanisms and combining them with potent β-lactam agents has been a successful therapeutic strategy to overcome resistance (5, 6).

Taniborbactam is a novel cyclic boronate-based β-lactamase inhibitor with inhibitory activity against the diversity of β-lactamases that impose significant clinical challenges to patient care, such as ESBL, cephalosporinases, KPC, and OXA-48 (7). Furthermore, unlike other novel β-lactamase inhibitors, it demonstrates activity against MBL (8). When combined with the fourth-generation cephalosporin cefepime, a zwitterion that readily penetrates to its active site within the periplasmic space of Gram-negative pathogens, cefepime-taniborbactam exhibits potent *in vitro* and *in vivo* antibacterial activity against Gram-negative clinical isolates exhibiting resistance to third-generation cephalosporins and carbapenems in Enterobacterales and *Pseudomonas aeruginosa* (9–12). In a randomized, double-blind, double dummy, Phase 3 clinical study of adult patients with complicated urinary tract infections (cUTI) or acute pyelonephritis, treatment with a 2-hour infusion of 2 g cefepime/0.5 g taniborbactam every 8 hours was non-inferior to and met the criteria for superiority compared to a 30-minute infusion of 1 g meropenem every 8 hours (13). The frequency of serious adverse events was comparable between the two treatment groups (13). These findings show promise of cefepime-taniborbactam for the treatment of cUTI caused by resistant Gram-negative pathogens (14).

Antimicrobial susceptibility testing is a vital component of selecting appropriate antimicrobial therapy for patient care. Establishing a standardized reference method that accommodates intra- and inter-laboratory variability ensures that clinical microbiology laboratories can provide reliable susceptibility testing results (15). In this study, establishing the reference method for cefepime-taniborbactam is described using the broth microdilution MIC assay conforming to the Clinical Laboratory Standards Institute M07 standard and International Standards Organization (ISO) standard 20776-1:2019.

## MATERIALS AND METHODS

### Bacterial isolates

Four of the six QC strains tested in the M23 tier 2 study are recommended in CLSI M100 for routine QC testing of certain β-lactam combination agents (16). The four strains consist of *E. coli* NCTC 13353 (CTX-M-15; ESBL-producing), *E. coli* ATCC 35218 (TEM-1; non-ESBL β-lactamase-producing), *K. pneumoniae* ATCC 700603 (SHV-18 and OXA-2 genotype; ESBL-producing), and *K. pneumoniae* ATCC BAA-1705 (KPC-2, TEM and SHV; carbapenemase producing). The other two isolates tested, *E. coli* ATCC 25922 (β-lactamase negative) and *P. aeruginosa* ATCC 27853 (PDC-5; inducible cephalosporinase producing), are not recommended as QC strains for routine QC of β-lactam combination agents (16).

Ten isolates were tested to assess the impact of broth microdilution medium or parameters on QC ranges or MIC results relative to the standard conditions. The isolates consisted of the six QC strains noted above, plus *E. coli* ATCC BAA-2814, and clinical isolates *E. coli* IHMA 1480076 (CMY-2, cephalosporinase producing), *K. pneumoniae* IHMA 1434760 (OXA-48; carbapenemase producing), and *K. pneumoniae* IHMA 1266420 (CTX-M-15, SHV, TEM; ESBL producing), which were obtained from a surveillance program.

### Broth microdilution MIC testing

Broth microdilution MIC testing in all laboratories participating in this study followed the procedures outlined in CLSI M07 standard and ISO standard 20776-1:2019 (17, 18). Unless indicated otherwise, bacterial inoculum suspensions were prepared in physiological saline, and optical density was measured using a spectrophotometer at a wavelength of 600 nm. Suspensions were adjusted to an optical density value calibrated to obtain a bacterial titer of 1–2 × 10^8^ CFU/mL and then diluted to obtain the equivalent of a 0.5 McFarland standard (5 × 10^5^ CFU/mL) as a starting inoculum density for the broth microdilution MIC assay.

### CLSI M23 tier 2 QC range study design

Performance of the M23 tier 2 study followed the recommended guidelines (19). In brief, nine qualified clinical laboratories performed 30 independent MIC determinations in three lots of cation-adjusted Mueller Hinton broth (CAMHB) obtained from three manufacturers over at least three days. The nine laboratories were as follows: Clinical Microbiology Institute, Wilsonville, OR; International Health Management Associates (IHMA), Schaumburg, IL; JMI Laboratories, North Liberty, IA; Laboratory Specialists, Inc., Westlake, OH; Micromyx, Kalamazoo, MI; Thermo Fisher Scientific, Oakwood Village, OH; University Hospital Cleveland Medical Center, Cleveland, OH; University of Rochester Medical Center, Rochester, NY; and Wake Forest University, Winston-Salem, NC. The three lots of CAMHB were from Difco (lot number 5181782; Becton Dickinson, Franklin Lakes, NJ), BD BBL (lot number 5257869; Becton Dickinson), and Oxoid (lot number 1433705; Thermo Fisher Scientific, Waltham, MA). A total of 270 MIC values (10 replicates × three media lots x nine sites) for each QC strain were determined. Broth microdilution MIC panels were manufactured by Thermo Fisher Scientific (Oakwood Village, OH) and were shipped and stored frozen until use. All reagents, media, and panels were used within their respective expiration dates.

### Broth microdilution QC testing during a surveillance study and the phase 3 clinical trial

Quality control MIC testing was performed concurrently during testing of clinical isolates from a 2018–2022 global surveillance study (International Health Management Associates, Inc., Schaumburg, IL) and patient isolates from the CERTAIN-1 study at two central microbiology laboratories (Labcorp, Indianapolis, IN, and Labcorp, Shanghai, China) (10, 13). Bacterial inoculum suspensions were prepared in physiological saline at the two central microbiology laboratories by determining the transmittance (450 nm) or absorbance (550 nm) using either a colorimeter or densitometer, respectively. Verification of colony counts was performed with *E. coli* ATCC 25922 on a quarterly basis at each site to confirm that the device readings maintained the target inoculum density of 1–2 × 10^8^ CFU/mL (16). Bacterial inoculum suspensions were diluted in CAMHB to inoculate the broth microdilution MIC panels (Thermo Fisher Scientific) as described above.

### Analysis of QC reference ranges

MIC data were evaluated as described in M23-A4 using RangeFinder (RangeFinder MIC XL 2003 and later.xls and RangeFinder DISK 2003 and later.xls) (19). As described in M23-A4, the proposed ranges may have included expansion of a range based on a “shoulder” of ≥60% of data points at the mode (19). Outlier data were determined by RangeFinder.

### Variation of MIC assay parameters

The standard broth microdilution MIC conditions require CAMHB containing 25 µg/mL of calcium and 12.5 µg/mL magnesium, pH of 7.2, a target inoculum density of 5 × 10^5^ CFU/mL (range 2–8 × 10^5^ CFU/mL), and incubation at 35 ± 2°C for 16–20 hours in ambient air (17, 20). Variations to CAMHB included altering the media pH (5.0, 6.0, and 8.0), adjustment of calcium (10 to 50 µg/mL; Sigma Aldrich, Inc., St. Louis, MO) or magnesium (10 to 50 µg/mL; Sigma Aldrich) cation content, and addition of 0.002% polysorbate-80 (Tween 80; Merck & Cie, Buchs, Switzerland), 1 mg/mL pulmonary surfactant (Survanta, Abbvie, St-Laurent, Canada), 50% pooled human serum (Innovative Research, Inc., Novi, MI), or 40 mg/mL human serum albumin (Sigma Aldrich). The inhibitory effects of pulmonary surfactant on daptomycin MIC determinations for *Staphylococcus aureus* ATCC 29213 and *Enterococcus faecalis* ATCC 29212 were used as positive controls (21). Assay parameter variations consisted of adjusting bacterial inoculum concentration (5 × 10^4^ CFU/mL, 5 × 10^6^ CFU/mL, and 5 × 10^7^ CFU/mL), incubation temperature (30°C or 40°C), and incubation duration (18, 20, 24, and 48 hours). For each variation, MIC testing was performed in triplicate using independent inoculum suspensions to obtain a modal MIC value.

## RESULTS

The M23 tier 2 study included nine laboratories to assess the intra- and inter-laboratory variability when establishing the QC ranges for the selected QC strains. All data were included in the analysis as no outlier data were observed using the Rangefinder program. Cefepime-taniborbactam MIC results for *E. coli* NCTC 13353 are shown in Table 1. The intra-laboratory MIC variability was low for *E. coli* NCTC 13353 as cefepime-taniborbactam MIC results spanned 2 to 3 log_2_ dilutions. The inter-laboratory variability was also low as the modal MIC values for each laboratory were either 0.25/4 µg/mL or 0.5/4 µg/mL, representing 91.5% (247/270) of all values. Due to the bimodal nature of the MIC results, a 4-dilution QC range of 0.12/4 to 1/4 µg/mL was determined, which encompassed 99.6% (269/270) of the MIC values (Table 2). Cefepime-taniborbactam QC ranges spanned three dilutions for *K. pneumoniae* ATCC BAA-1705 (Table S1), *K. pneumoniae* ATCC 700603 (Table S2), *E. coli* ATCC 35218 (Table S3), and *E. coli* ATCC 25922 (Table S4). For *P. aeruginosa* ATCC 27853, a bimodal distribution of the MIC results with >60% of the data points at each mode was observed, resulting in a QC range of four dilutions (Table S5).

**TABLE 1:** Summary of M23 tier 2 study results for cefepime-taniborbactam broth microdilution MICs obtained for *E. coli* NCTC 13353 stratified by CAMHB media lots or by participating laboratories^*a*^

Cefepime-taniborbactam MIC (µg/mL)^*b*^	Number of occurrences at MIC by CAMHB lot			Number of occurrences at MIC by laboratory									
	Difco	Becton Dickinson	Oxoid	1	2	3	4	5	6	7	8	9	Total
0.12/4	6		2					1	6			1	8
0.25/4	54	32	27	11	11	5		18	18	6	18	26	113
0.5/4	27	52	55	19	18	22	19	11	6	24	12	3	134
1/4	2	6	6		1	3	10						14
2/4	1						1						1
Total	90	90	90	30	30	30	30	30	30	30	30	30	270
Mode	0.25	0.5	0.5	0.5	0.5	0.5	0.5	0.25	0.25	0.5	0.25	0.25	0.5
Geometric mean	0.31	0.41	0.41	0.39	0.40	0.48	0.66	0.31	0.25	0.44	0.33	0.26	0.37
Dilution range	5	3	4	2	3	3	3	3	3	2	2	3	5

^*a*^ The empty cells indicate the absence of occurrences of an MIC at a particular cefepime-taniborbactam MIC.

^*b*^ The shaded area depicts MIC values within the cefepime-taniborbactam CLSI-approved QC range for *E. coli* NCTC 13353 (22).

**TABLE 2:** Summary of broth microdilution MIC QC strain testing results with cefepime-taniborbactam (fixed at 4 µg/mL) and approved CLSI QC ranges

	Cefepime-taniborbactam			Cefepime		
QC strain	QC range	Number of doubling dilutions in range	% (n/N) of M23 tier 2 MIC values in range	QC range	Number of doubling dilutions in range	% (n/N) of M23 tier 2 MIC values in range
Strain recommended for routine QC testing						
*E. coli* NCTC 13353	0.12/4–1/4	4	99.6 (269/270)	≥64	–^*a*^	100 (90/90)
Alternate QC strain						
*K. pneumoniae* ATCC BAA-1705	0.12/4–0.5/4	3	98.9 (267/270)	–	–	–
Strains not recommended for QC testing						
*E. coli* ATCC 25922	0.03/4–0.12/4	3	100 (270/270)	0.016–0.12	4	100 (90/90)
*E. coli* ATCC 35218	0.016/4–0.06/4	3	100 (270/270)	0.008–0.06	4	97.8 (88/90)
*K. pneumoniae* ATCC 700603	0.12/4–0.5/4	3	99.3 (268/270)	0.5–2	3	98.9 (89/90)
*P. aeruginosa* ATCC 27853	0.5/4–4/4	4	100 (270/270)	0.5–4	4	98.9 (89/90)

^*a*^ “–”, Not available.

The cefepime-taniborbactam QC MIC results for *E. coli* NCTC 13353, obtained during testing of clinical isolates as part of a 2018–2022 global surveillance study and at the two central microbiology laboratories of the CERTAIN-1 Phase 3 clinical study, are shown in Table 3. The modal MIC values obtained were 0.25/4 µg/mL, and 98.9% (177/179) of the QC results occurred within the established QC range. These values are comparable to those of the M23 tier 2 study, demonstrating the reproducibility of the assay.

**TABLE 3:** Summary of cefepime-taniborbactam MIC QC results obtained for *E. coli* NCTC 13353 performed during a surveillance study and at the central microbiology laboratories participating in the CERTAIN-1 Phase 3 clinical study

Cefepime-taniborbactam MIC (µg/mL)^*a*^	Number of occurrences at MIC				
	Surveillance^*b*^	Central laboratory in CERTAIN-1^*c*^			Total
		Site 1	Site 2	Combined	
0.06/4	0	2	0	2	2
0.12/4	4	3	0	3	7
0.25/4	45	59	14	73	118
0.5/4	27	20	2	22	49
1/4	3	0	0	0	3
2/4	0	0	0	0	0
Total	79	84	16	100	179
Mode	0.25/4	0.25/4	0.25/4	0.25/4	0.25/4
% in range	100	97.6	100	98.0	98.9
Dilution range	4	4	2	4	5

^*a*^ The shaded area depicts MIC values within the cefepime-taniborbactam CLSI-approved QC range for E. coli NCTC 13353 (22).

^*b*^ Reference (10).

^*c*^ Reference (13).

To determine if variations to standardized CAMHB could affect cefepime-taniborbactam susceptibility testing results, changes to pH, cation content, or supplementation with polysorbate-80, pulmonary surfactant, human serum, or human serum albumin were assessed. In general, most MIC values relative to standard conditions were unchanged, except for some instances of minor MIC shifts. Varying the pH, cation content (Ca^2+^ or Mg^2+^), or addition of 0.002% polysorbate-80, 50% human serum or 40 mg/mL HSA did not cause cefepime-taniborbactam modal MIC results to shift outside of the established QC ranges for *E. coli* NCTC 13353 or any of the other 5 QC strains, except for higher cefepime modal MIC values for *K. pneumoniae* ATCC 700603 at pH 5 to 6 (Table 4). For the other tested isolates, variations to CAMHB did cause some instances of isolate-specific effects on cefepime and cefepime-taniborbactam modal MIC values, but no specific causal pattern was observed (Table 4). While the addition of 1 mg/mL pulmonary surfactant increased the daptomycin MIC by ≥32-fold against the QC strains *S. aureus* ATCC 29213 and *E. faecalis* ATCC 29212 that were used as positive controls (Table S6), cefepime and cefepime-taniborbactam modal MIC values for the 10 tested isolates in the presence of surfactant were within twofold of modal MICs in its absence (Table 4).

**TABLE 4:** Effect of changes to standard CAMHB on cefepime and cefepime-taniborbactam broth microdilution MIC determinations^*a*^^,^^*b*^

Bacterial isolate	Major β-lactamase	Standard		pH						Ca^2+^ (µg/mL) supplementation				Mg^2+^ (µg/mL) supplementation				0.002% P-80		1 mg/mL pulmonary surfactant		50% human serum		40 mg/mL HSA	
				5.0		6.0		8.0		10		50		10		50									
		FEP	FTB	FEP	FTB	FEP	FTB	FEP	FTB	FEP	FTB	FEP	FTB	FEP	FTB	FEP	FTB	FEP	FTB	FEP	FTB	FEP	FTB	FEP	FTB
QC strains																									
*E. coli* NCTC 13353	CTX-M-15	>64	0.25	>64	0.5	>64	0.5	**32**	0.5	>64	0.5	>64	0.25	>64	0.5	>64	0.25	>64	0.5	64	0.25	NA^*c*^	NA	>64	0.5
*K. pneumoniae* BAA-1705	KPC-2	>64	0.25	64	0.5	>64	0.25	32	0.25	64	0.12	>64	0.12	32	0.12	32	0.25	64	0.25	64	0.25	>64	0.12	>64	0.12
*K. pneumoniae* ATCC 700603	SHV-18	0.5	0.25	**8**	0.5	**4**	0.5	0.5	0.12	0.5	0.25	0.5	0.12	1	0.25	0.5	0.25	1	0.25	1	0.25	**0.06**	**0.06**	1	0.25
*E. coli* ATCC 35218	TEM-1	0.016	0.016	0.06	0.06	0.06	0.03	0.03	0.016	0.03	0.016	0.03	0.016	0.03	0.016	0.03	0.03	0.06	0.016	≤0.03	0.03	0.03	0.016	0.03	0.03
*E. coli* ATCC 25922	None	0.03	0.06	0.12	0.12	0.06	0.06	0.03	0.03	0.03	0.03	0.03	0.06	0.06	0.06	0.03	0.06	0.06	0.06	≤0.03	0.03	0.03	0.03	0.06	0.06
*P. aeruginosa* ATCC 27853	PDC-5	1	1	2	2	2	2	2	2	1	1	1	1	1	1	1	1	2	2	1	2	0.5	0.5	1	1
Other isolates																									
*K. pneumoniae* BAA-2814	KPC-3	>64	1	>64	4	>64	2	>64	2	>64	1	>64	1	>64	1	>64	1	>64	1	>64	1	>64	0.5	>64	0.5
*E. coli* IHMA 1480076	CMY-2	0.25	0.03	0.5	0.06	0.5	0.06	0.25	0.06	0.5	0.03	0.25	0.03	0.25	0.06	0.25	0.06	0.5	0.06	0.25	0.06	NA	NA	0.25	0.12
*K. pneumoniae* IHMA 1266420	CTX-M-15	>64	0.5	>64	2	>64	1	32	0.5	>64	0.5	>64	0.5	>64	0.5	>64	0.5	64	0.5	64	0.5	>64	0.25	>64	0.5
*K. pneumoniae* IHMA 1434760	OXA-48	>64	1	>64	2	>64	1	>64	2	>64	1	>64	1	>64	1	>64	1	>64	1	>64	1	>64	0.5	>64	0.5

^*a*^ Abbreviations: FEP, cefepime; FTB, cefepime-taniborbactam; HSA, human serum albumin; P-80, polysorbate-80.

^*b*^ Cefepime-taniborbactam MIC values are shown without designation of the fixed concentration of 4 µg/mL taniborbactam (e.g., x/4). MIC values are in bold for QC strains when a variation to CAMHB caused the determined MIC to be outside of the CLSI-approved QC range (22). MIC values are underlined when a variation to CAMHB caused a ≥4-fold shift relative to the MIC value determined using standard conditions.

^*c*^ "NA", Not available.

When assessing the effect of varying assay parameters on MIC results, increasing the bacterial inoculum density had the greatest effect. Specifically, increasing the bacterial inoculum tenfold to 5 × 10^6^ CFU/mL increased the modal MIC value by 32-fold to 8/4 µg/mL for *E. coli* NCTC 13353 and by fourfold to 1/4 µg/mL for *K. pneumoniae* ATCC BAA-1705. Changes to other assay parameters, such as incubation temperature and duration, had mostly negligible effects on the tested isolates, although an incubation time of 48 hours increased the cefepime-taniborbactam modal MIC values by fourfold within the established QC ranges for *E. coli* NCTC 13353 and *P. aeruginosa* ATCC 27853 (Table 5).

**TABLE 5:** Effect of changes to standard broth microdilution MIC assay conditions on cefepime and cefepime-taniborbactam broth microdilution MIC determinations^*a*^

		MIC^*b*^ (µg/mL)																			
Bacterial isolate	Main β-lactamase	Standard		Bacterial inoculum						Incubation temperature						Incubation time					
				5 × 10^4^ CFU/ml		5 × 10^6^ CFU/ml		5 × 10^7^ CFU/ml		30°C		40°C		18 h		20 h		24 h		48 h	
		FEP	FTB	FEP	FTB	FEP	FTB	FEP	FTB	FEP	FTB	FEP	FTB	FEP	FTB	FEP	FTB	FEP	FTB	FEP	FTB
QC strains																					
*E. coli* NCTC 13353	CTX-M-15	>64	0.25	**32**	0.25	>64	**8**	>64	**>64**	>64	0.5	64	0.5	>64	0.25	>64	0.5	>64	0.5	>64	1
*K. pneumoniae* BAA-1705	KPC-2	>64	0.25	**8**	0.12	>64	**1**	>64	**>64**	64	0.25	**32**	0.12	>64	0.25	>64	0.25	>64	0.25	>64	0.25
*K. pneumoniae* ATCC 700603	SHV-18	0.5	0.25	0.5	0.25	32	0.25	**>64**	**4**	1	0.25	0.5	0.25	1	0.25	1	0.25	1	0.25	1	0.25
*E. coli* ATCC 35218	TEM-1	0.016	0.016	0.03	0.016	0.06	0.03	**4**	**2**	0.03	0.03	0.03	0.016	0.03	0.03	0.06	0.03	0.06	0.06	0.06	0.06
*E. coli* ATCC 25922	None	0.03	0.06	0.06	0.03	0.06	0.06	**2**	**2**	0.06	0.06	0.06	0.06	0.03	0.06	0.03	0.06	0.06	0.06	0.06	0.06
*P. aeruginosa* ATCC 27853	PDC-5	1	1	1	1	2	2	**>64**	**32**	1	1	1	1	1	1	1	2	2	2	4	4
Other isolates																					
*K. pneumoniae* BAA-2814	KPC-3	>64	1	64	1	>64	1	>64	64	>64	1	>64	1	>64	1	>64	1	>64	1	>64	1
*E. coli* IHMA 1480076	CMY-2	0.25	0.03	0.25	0.03	1	0.03	16	4	ND^*c*^	ND	ND	ND	ND	ND	ND	ND	ND	ND	ND	ND
*K. pneumoniae* IHMA 1266420	CTX-M-15	>64	0.5	64	0.25	>64	8	>64	>64	>64	0.5	64	0.5	>64	0.5	>64	0.5	>64	0.5	>64	0.5
*K. pneumoniae* IHMA 1434760	OXA-48	>64	1	>64	1	>64	1	>64	8	>64	1	>64	0.5	>64	1	>64	1	>64	1	>64	1

^*a*^ Abbreviations: FEP, cefepime; FTB, cefepime-taniborbactam.

^*b*^ MIC values are shown without the designation of the fixed concentration of taniborbactam (e.g., x/4). MIC values in bold are for QC strains when a change to standard assay conditions caused the determined MIC to be outside of the CLSI-approved QC range (22). MIC values are underlined when a change in the standard assay condition caused a ≥4-fold shift relative to the MIC value determined under standard conditions.

^*c*^ "ND", Not determined.

## DISCUSSION

Developing an AST reference method requires (1) establishing QC ranges for carefully selected QC strains and (2) ensuring that the assay is robust and can accommodate inter-laboratory variability arising from handling by different laboratory personnel, technical factors, and brands of CAMHB (15). Assessing the impact of varying these components is vital to ascertaining that appropriate assay performance can be attained in clinical microbiology laboratories. In the M23 tier 2 study, cefepime-taniborbactam MIC results from all nine participating laboratories were used to establish the QC ranges for *E. coli* NCTC 13353 (and the other tested QC strains), as none of the data generated were deemed to be statistical outliers. The cefepime-taniborbactam 4-log_2_ dilution QC range determined for *E. coli* NCTC 13353 exceeded the 3-log_2_ dilution ranges set for other cefepime-β-lactamase inhibitor combinations in CLSI M100^16^. Although MIC results spanned 2 to 3-log_2_ dilutions in each of the nine laboratories, four laboratories reported a modal MIC of 0.25/4 µg/mL, whereas the other five laboratories reported a modal MIC of 0.5/4 µg/mL. Furthermore, one of the tested brands of CAMHB (Difco) yielded a twofold lower modal MIC value of 0.25/4 µg/mL relative to the other two brands (Becton Dickinson, Oxoid). When considering QC data from all 12 laboratories (i.e., the M23 tier 2 study, surveillance, and the two central laboratories of the phase 3 trial), 99.3% (446/449) of the combined QC values were within the 4-dilution QC range. Thus, the extent of testing, which comprised numerous qualified laboratory personnel, CAMHB lots sourced from different manufacturers, and a testing timeframe of 5 years, provides assurance that the CLSI 4-dilution QC range for cefepime-taniborbactam better encompasses the expected testing variability for this strain when the reference method is performed according to the CLSI standard (16, 17).

None of the modifications to the standardized assay conditions or CAMHB other than increasing the starting inoculum caused systematic changes with all tested QC strains and additional isolates. Supplementation of CAMHB with 50 µg/mL Ca^2+^ or Mg^2+^ increased the cefepime-taniborbactam modal MIC of *K. pneumoniae* ATCC BAA-1705; however, these cation concentrations are ≥2-fold the amount of 20–25 µg/mL Ca^2+^ and 10–12.5 µg/mL Mg^2+^ stipulated in the CLSI M07 standard (17). Thus, minor variations are not expected to affect susceptibility determinations using the broth microdilution MIC reference method. The lack of effect of biological components, such as human serum, albumin, or pulmonary surfactant, *in vitro* predicts that cefepime-taniborbactam will retain antibacterial activity within these matrices *in vivo*. Due to the known inoculum effect that has been described for cefepime and other β-lactams (23, 24), increasing the inoculum density to 5 × 10^7^ CFU/mL increased cefepime and cefepime-taniborbactam modal MIC values for all the tested organisms. Moreover, consistent with a previous report describing the sensitivity of cefepime MIC values to discreet inocula (25), a starting inoculum of 5 × 10^6^ CFU/mL also caused an elevated cefepime-taniborbactam MIC for *E. coli* NCTC 13353 and *K. pneumoniae* ATCC BAA-1705, reinforcing that an inoculum within the CLSI standardized range of 2–8 × 10^5^ CFU/mL is important for obtaining accurate MIC results. The CLSI recommendation to perform weekly inoculum colony counts will help ensure that the inoculum is appropriate using the standardized method (17).

The ongoing development of beta-lactam/beta-lactamase inhibitor combinations has been accompanied by an increased number of QC strains (22). Despite the corresponding increased complexity of routine testing, it is important for users to recognize that unless a strain has been designated for routine QC (as indicated in M100^16^), it may be of *no* value to control the beta-lactam and beta-lactamase inhibitor simultaneously. Although cefepime-taniborbactam QC ranges were established for six QC strains, *E. coli* NCTC 13353 was the only strain with non-overlapping QC ranges for cefepime and cefepime-taniborbactam. Therefore, it simultaneously controls appropriate antibacterial activity of cefepime and β-lactamase inhibition by taniborbactam. Accordingly, it is the only strain recommended for routine QC testing of cefepime-taniborbactam in CLSI M100 as of Edition 30 (2020). Furthermore, *E. coli* NCTC 13353 can also alert end users to the potential root cause of QC failures, either elevated pH or inoculum effect, to aid in their quality assurance investigations. *Klebsiella pneumoniae* ATCC BAA-1705 is an alternate QC strain, albeit a QC range for cefepime is not available, and imipenem is needed to assess the integrity of KPC production. It is important to emphasize that the QC strains *E. coli* ATCC 25922, *P. aeruginosa* ATCC 27853, *E. coli* ATCC 35218, and *K. pneumoniae* ATCC 700603 are of no value in controlling for the presence and activity of both the β-lactam and β-lactamase inhibitor because the overlapping QC ranges observed for cefepime and cefepime-taniborbactam could mistakenly lead to the assumption that taniborbactam is present and active when indeed it may not be. Consequently, the use of these QC strains in a setting of degraded (or absent) taniborbactam could subsequently lead to an unrecognized interpretive error of false resistance of the patient isolate in which the treating clinician is misinformed as to the potential clinical utility of cefepime-taniborbactam. In light of this, *E. coli* NCTC 13353 is recommended as the routine QC strain for cefepime-taniborbactam, based on its reliable and discriminatory performance as described here and its use as a routine QC strain for certain other β-lactam/β-lactamase combinations (22).

## SUPPLEMENTAL MATERIAL

Supplemental tables jcm.00661-25-s0001.docx Tables S1 to S6.

ASM does not own the copyrights to Supplemental Material that may be linked to, or accessed through, an article. The authors have granted ASM a non-exclusive, world-wide license to publish the Supplemental Material files. Please contact the corresponding author directly for reuse.
