# Supplementary material for: Establishing the reference broth microdilution MIC method for cefepime-taniborbactam
Source: J Clin Microbiol. 2025 Aug 19;63(9):e00661-25. doi: 10.1128/jcm.00661-25 (PMC12421830; doi:10.1128/jcm.00661-25)
Supplement: Supplemental tables — Tables S1 to S6. [file jcm.00661-25-s0001.docx]

Supplemental material

Establishing the Reference Broth Microdilution MIC Method for Cefepime-Taniborbactam

Adam Belley^1^, Susan M. Cusick^1^, Dan C. Pevear^1^, Laura Koeth^2^, Jeanna DiFranco-Fisher^2^, Nimmi Kothari^3^, Stephen Hawser^3^, Greg Moeck^1^

^1^Venatorx Pharmaceuticals, Inc., Malvern, PA

^2^Laboratory Specialists, Inc., Westlake, OH

^3^International Health Management Associates Europe Sàrl, Monthey, Switzerland

Supplemental Table 1. Summary of M23 tier 2 study results for cefepime-taniborbactam broth microdilution MICs obtained for *K. pneumoniae* ATCC BAA-1705 stratified by CAMHB media lots or by participating laboratories.

| Cefepime-taniborbactam MIC (µg/mL)^1^ | Number of occurrences at MIC  by CAMHB lot | | |  | Number of occurrences at MIC by laboratory | | | | | | | | | | |
| --- | --- | --- | --- | --- | --- | --- | --- | --- | --- | --- | --- | --- | --- | --- | --- |
|  | Difco | Becton Dickinson | Oxoid |  | 1 | 2 | 3 | 4 | 5 | 6 | 7 | 8 | 9 | Total |  |
| 0.03/4 |  |  |  |  |  |  |  |  |  |  |  |  |  |  |  |
| 0.06/4 |  |  |  |  |  |  |  |  |  |  |  |  |  |  |  |
| 0.12/4 | 4 |  | 3 |  |  |  | 2 | 1 | 1 | 2 |  |  | 1 | 7 |  |
| 0.25/4 | 79 | 79 | 73 |  | 27 | 24 | 22 | 26 | 26 | 28 | 27 | 26 | 25 | 231 |  |
| 0.5/4 | 6 | 9 | 14 |  | 3 | 6 | 6 | 1 | 3 |  | 2 | 4 | 4 | 29 |  |
| 1/4 | 1 | 2 |  |  |  |  |  | 2 |  |  | 1 |  |  | 3 |  |
| 2/4 |  |  |  |  |  |  |  |  |  |  |  |  |  |  |  |
| Total | 90 | 90 | 90 |  | 30 | 30 | 30 | 30 | 30 | 30 | 30 | 30 | 30 | 270 |  |
| Mode | 0.25 | 0.25 | 0.25 |  | 0.25 | 0.25 | 0.25 | 0.25 | 0.25 | 0.25 | 0.25 | 0.25 | 0.25 | 0.25 |  |
| Geometric mean | 0.26 | 0.28 | 0.27 |  | 0.27 | 0.29 | 0.27 | 0.27 | 0.26 | 0.24 | 0.27 | 0.27 | 0.27 | 0.27 |  |
| Dilution range | 4 | 3 | 3 |  | 2 | 2 | 3 | 4 | 3 | 2 | 3 | 2 | 3 | 4 |  |

^1^The shaded area depicts MIC values within the cefepime-taniborbactam CLSI-approved QC range for *K. pneumoniae* ATCC BAA-1705^25^.

Supplemental Table 2. Summary of M23 tier 2 study results for cefepime-taniborbactam broth microdilution MICs obtained for *K. pneumoniae* ATCC 700603 stratified by CAMHB media lots or by participating laboratories.

| Cefepime-taniborbactam MIC (µg/mL)^1^ | Number of occurrences at MIC  by CAMHB lot | | |  | Number of occurrences at MIC by laboratory | | | | | | | | | | |
| --- | --- | --- | --- | --- | --- | --- | --- | --- | --- | --- | --- | --- | --- | --- | --- |
|  | Difco | Becton Dickinson | Oxoid |  | 1 | 2 | 3 | 4 | 5 | 6 | 7 | 8 | 9 | Total |  |
| 0.03/4 |  |  |  |  |  |  |  |  |  |  |  |  |  |  |  |
| 0.06/4 |  |  |  |  |  |  |  |  |  |  |  |  |  |  |  |
| 0.12/4 | 4 | 1 | 1 |  |  |  | 2 |  |  | 4 |  |  |  | 6 |  |
| 0.25/4 | 72 | 60 | 69 |  | 25 | 23 | 21 | 7 | 23 | 24 | 27 | 23 | 28 | 201 |  |
| 0.5/4 | 13 | 28 | 20 |  | 5 | 6 | 7 | 22 | 7 | 2 | 3 | 7 | 2 | 61 |  |
| 1/4 | 1 | 1 |  |  |  | 1 |  | 1 |  |  |  |  |  | 2 |  |
| 2/4 |  |  |  |  |  |  |  |  |  |  |  |  |  |  |  |
| Total | 90 | 90 | 90 |  | 30 | 30 | 30 | 30 | 30 | 30 | 30 | 30 | 30 | 270 |  |
| Mode | 0.25 | 0.25 | 0.25 |  | 0.25 | 0.25 | 0.25 | 0.5 | 0.25 | 0.25 | 0.25 | 0.25 | 0.25 | 0.25 |  |
| Geometric mean | 0.27 | 0.31 | 0.29 |  | 0.28 | 0.30 | 0.28 | 0.44 | 0.29 | 0.24 | 0.27 | 0.29 | 0.26 | 0.29 |  |
| Dilution range | 4 | 4 | 3 |  | 2 | 3 | 3 | 3 | 2 | 3 | 2 | 2 | 2 | 4 |  |

^1^The shaded area depicts MIC values within the cefepime-taniborbactam CLSI-approved QC range for *K. pneumoniae* ATCC 700603^25^.

Supplemental Table 3. Summary of M23 tier 2 study results for cefepime-taniborbactam broth microdilution MICs obtained for *E. coli* ATCC 35218 stratified by CAMHB media lots or by participating laboratories.

| Cefepime-taniborbactam MIC (µg/mL)^1^ | Number of occurrences at MIC  by CAMHB lot | | |  | Number of occurrences at MIC by laboratory | | | | | | | | | | |
| --- | --- | --- | --- | --- | --- | --- | --- | --- | --- | --- | --- | --- | --- | --- | --- |
|  | Difco | Becton Dickinson | Oxoid |  | 1 | 2 | 3 | 4 | 5 | 6 | 7 | 8 | 9 | Total |  |
| 0.008/4 |  |  |  |  |  |  |  |  |  |  |  |  |  |  |  |
| 0.016/4 | 21 | 5 | 3 |  |  | 1 | 5 |  | 8 | 14 | 1 |  |  | 29 |  |
| 0.03/4 | 61 | 76 | 79 |  | 30 | 25 | 23 | 25 | 22 | 15 | 25 | 26 | 25 | 216 |  |
| 0.06/4 | 8 | 9 | 8 |  |  | 4 | 2 | 5 |  | 1 | 4 | 4 | 5 | 25 |  |
| 0.12/4 |  |  |  |  |  |  |  |  |  |  |  |  |  |  |  |
| 0.25/4 |  |  |  |  |  |  |  |  |  |  |  |  |  |  |  |
| Total | 90 | 90 | 90 |  | 30 | 30 | 30 | 30 | 30 | 30 | 30 | 30 | 30 | 270 |  |
| Mode | 0.03 | 0.03 | 0.03 |  | 0.03 | 0.03 | 0.03 | 0.03 | 0.03 | 0.016 | 0.03 | 0.03 | 0.03 | 0.03 |  |
| Geometric mean | 0.03 | 0.03 | 0.03 |  | 0.03 | 0.03 | 0.03 | 0.03 | 0.03 | 0.023 | 0.03 | 0.03 | 0.03 | 0.03 |  |
| Dilution range | 3 | 3 | 3 |  | 1 | 3 | 3 | 2 | 2 | 3 | 3 | 2 | 2 | 3 |  |

^1^The shaded area depicts MIC values within the cefepime-taniborbactam CLSI-approved QC range for *E. coli* ATCC 35218^25^.

Supplemental Table 4. Summary of M23 tier 2 study results for cefepime-taniborbactam broth microdilution MICs obtained for *E. coli* ATCC 25922 stratified by CAMHB media lots or by participating laboratories.

| Cefepime-taniborbactam MIC (µg/mL)^1^ | Number of occurrences at MIC  by CAMHB lot | | |  | Number of occurrences at MIC by laboratory | | | | | | | | | | |
| --- | --- | --- | --- | --- | --- | --- | --- | --- | --- | --- | --- | --- | --- | --- | --- |
|  | Difco | Becton Dickinson | Oxoid |  | 1 | 2 | 3 | 4 | 5 | 6 | 7 | 8 | 9 | Total |  |
| 0.016/4 |  |  |  |  |  |  |  |  |  |  |  |  |  |  |  |
| 0.03/4 | 28 | 15 | 2 |  | 5 | 16 | 4 |  | 4 | 7 | 3 | 3 | 3 | 45 |  |
| 0.06/4 | 56 | 69 | 85 |  | 24 | 14 | 25 | 25 | 24 | 23 | 21 | 27 | 27 | 210 |  |
| 0.12/4 | 6 | 6 | 3 |  | 1 |  | 1 | 5 | 2 |  | 6 |  |  | 15 |  |
| 0.25/4 |  |  |  |  |  |  |  |  |  |  |  |  |  |  |  |
| 0.5/4 |  |  |  |  |  |  |  |  |  |  |  |  |  |  |  |
| Total | 90 | 90 | 90 |  | 30 | 30 | 30 | 30 | 30 | 30 | 30 | 30 | 30 | 270 |  |
| Mode | 0.06 | 0.06 | 0.06 |  | 0.06 | 0.03 | 0.06 | 0.06 | 0.06 | 0.06 | 0.06 | 0.06 | 0.06 | 0.06 |  |
| Geometric mean | 0.05 | 0.06 | 0.06 |  | 0.05 | 0.04 | 0.06 | 0.07 | 0.06 | 0.05 | 0.06 | 0.06 | 0.06 | 0.06 |  |
| Dilution range | 3 | 3 | 3 |  | 3 | 2 | 3 | 2 | 3 | 2 | 3 | 2 | 2 | 3 |  |

^1^The shaded area depicts MIC values within the cefepime-taniborbactam CLSI-approved QC range for *E. coli* ATCC 25922^25^.

Supplemental Table 5. Summary of M23 tier 2 study results for cefepime-taniborbactam broth microdilution MICs obtained for *P. aeruginosa* ATCC 27853 stratified by CAMHB media lots or by participating laboratories.

| Cefepime-taniborbactam MIC (µg/mL) | Number of occurrences at MIC  by CAMHB lot | | |  | Number of occurrences at MIC by laboratory | | | | | | | | | | |
| --- | --- | --- | --- | --- | --- | --- | --- | --- | --- | --- | --- | --- | --- | --- | --- |
|  | Difco | Becton Dickinson | Oxoid |  | 1 | 2 | 3 | 4 | 5 | 6 | 7 | 8 | 9 | Total |  |
| 0.25/4 |  |  |  |  |  |  |  |  |  |  |  |  |  |  |  |
| 0.5/4 |  |  |  |  |  |  |  |  |  |  |  |  |  |  |  |
| 1/4 | 48 | 33 | 35 |  | 10 | 14 | 7 | 14 | 23 | 24 | 4 | 17 | 3 | 116 |  |
| 2/4 | 38 | 53 | 47 |  | 16 | 15 | 23 | 14 | 7 | 6 | 25 | 8 | 24 | 138 |  |
| 4/4 | 4 | 4 | 8 |  | 4 | 1 |  | 2 |  |  | 1 | 5 | 3 | 16 |  |
| 8/4 |  |  |  |  |  |  |  |  |  |  |  |  |  |  |  |
| Total | 90 | 90 | 90 |  | 30 | 30 | 30 | 30 | 30 | 30 | 30 | 30 | 30 | 270 |  |
| Mode | 1 | 2 | 2 |  | 2 | 2 | 2 | 2 | 1 | 1 | 2 | 1 | 2 | 2 |  |
| Geometric mean | 1.4 | 1.6 | 1.6 |  | 1.7 | 1.5 | 1.7 | 1.5 | 1.2 | 1.1 | 1.9 | 1.5 | 2.0 | 1.5 |  |
| Dilution range | 3 | 3 | 3 |  | 3 | 3 | 2 | 3 | 2 | 2 | 3 | 3 | 3 | 3 |  |

^1^The shaded area depicts MIC values within the cefepime-taniborbactam CLSI-approved QC range for *P. aeruginosa* ATCC 27853^25^.

Supplemental Table 6. Effect of 1 mg/mL of pulmonary surfactant on daptomycin MIC value for control isolates

| Strain | Daptomycin MIC (µg/mL) | |
| --- | --- | --- |
|  | Standard conditions | with 1 mg/mL  pulmonary surfactant |
| *S. aureus* ATCC 29213 | 1 | 128 |
| *E. faecalis* ATCC 29212 | 4 | 128 |
| *E. coli* ATCC 25922 | >128 | >128 |
| *P. aeruginosa* ATCC 27853 | >128 | >128 |
